# Supplementary material for: An electrical analogy to Mie scattering
Source: Nat Commun. 2016 Sep 27;7:12894. doi: 10.1038/ncomms12894 (PMC5052640; doi:10.1038/ncomms12894)
Supplement: Supplementary Information — Supplementary Figures 1-11, Supplementary Table 1, Supplementary Notes 1-6, Supplementary Methods and Supplementary References. [file ncomms12894-s1.pdf]

Supplementary Figures

Supplementary Figure 1

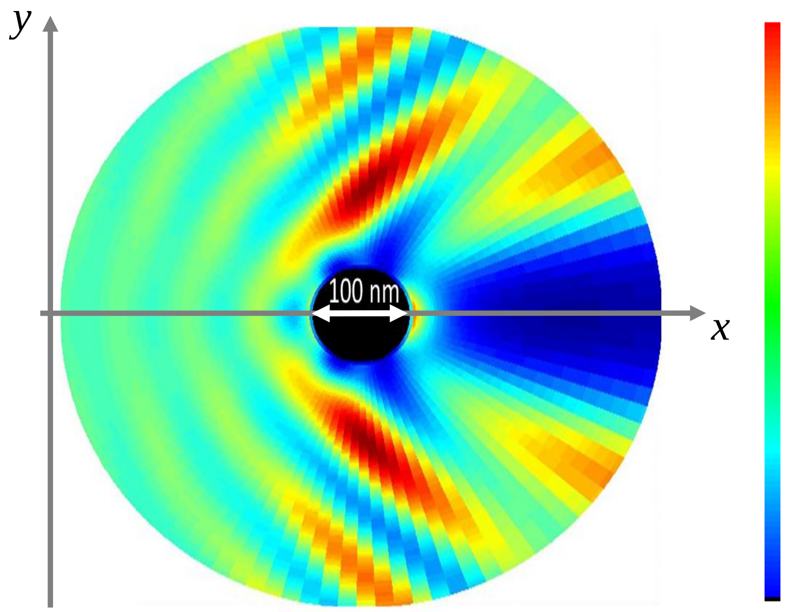

Supplementary Figure 1. Calculated electronic density distribution outside a circular potential scatterer. The diameter of the scatterer is 100 nm with a potential magnitude of V_{pot} = -0.12 eV, V_{gate} = +3 V (red/blue indicate high/low density).

Supplementary Figure 2

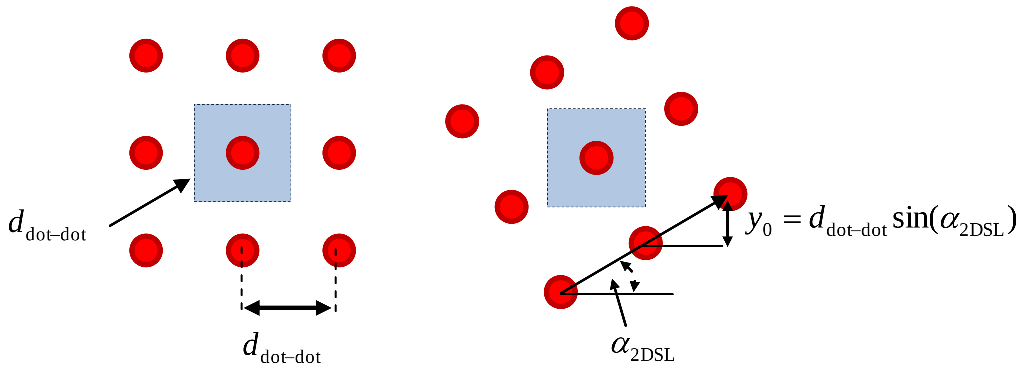

Supplementary Figure 2. Illustration of the building block considered to construct the non-canted (left) and canted (right) 2DSLs. Also included are the relevant parameters such as $y_0 = d_{\text{dot-dot}} \sin(\alpha_{\text{2DSL}})$, which provides the transversal coordinate of the centre of the different circular scattering potentials.

Supplementary Figure 3

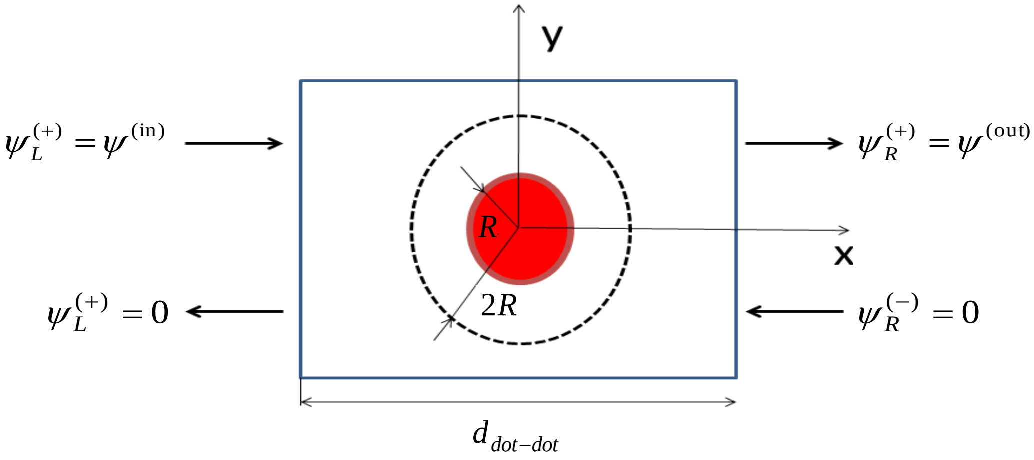

Supplementary Figure 3. Illustration of a block from a transfer matrix method viewpoint containing one single scattering potential (dot). $2R$ represents the far-field limit in this situation which is smaller than the spacing of two neighbouring dot-centres ($d_{\text{dot-dot}}$).

Supplementary Figure 4

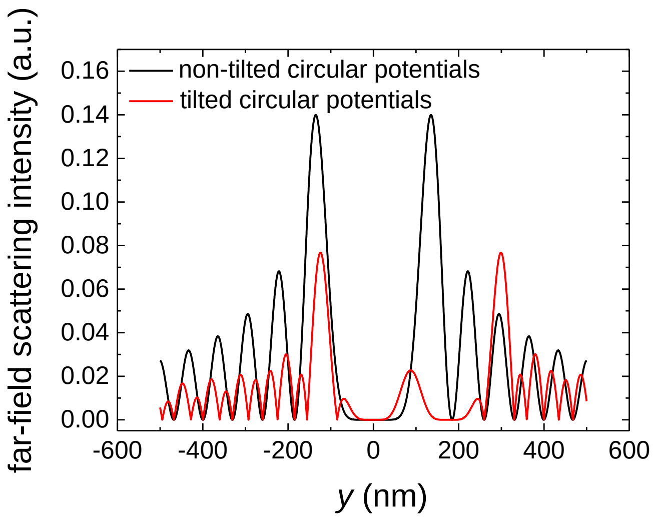

Supplementary Figure 4. Calculated transversal electronic far-field scattering intensity for two circular potentials. The two potentials are placed in a non-canted (black) and canted (red) configuration with respect to the incoming wavefunction. A spatially imbalanced intensity appears in the canted case. In both cases, V_{pot}= -0.12 eV, ΔV_{gate}= +1V.

Supplementary Figure 5

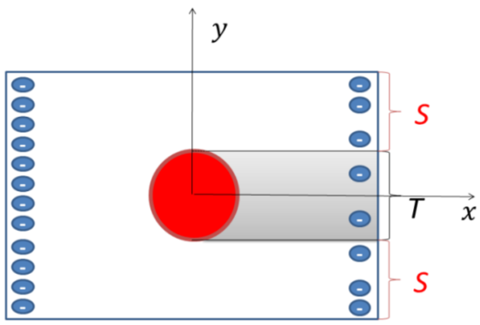

Supplementary Figure 5. **Evaluation of the far-field transmitted T and scattered S probabilities**. This is applicable in the far-field limit for the case of Dirac-electrons within a single-dot block.

Supplementary Figure 6

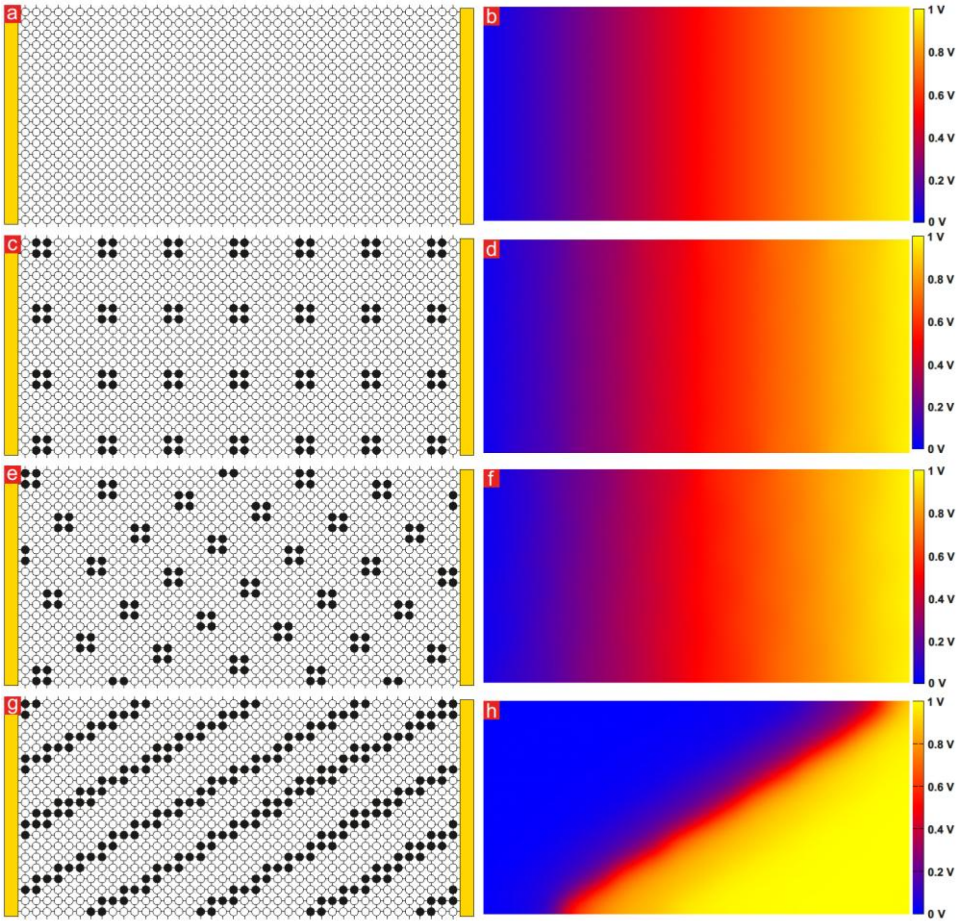

Supplementary Figure 6. Strictly diffusive (non-relativistic) resistor networks without and with imposed scattering-potential 2DSL. In the left column (**(a)**, **(c)**, **(e)**, **(g)**) the network is shown including the imposed 2DSL. The resulting potential landscape is shown in the right column (**(b)**, **(d)**, **(f)**, **(h)**). In both cases a driving voltage of 1 V is applied. The network consists of 20 x 40 four-terminal resistors (discs), the (local) scattering potentials are black. No transverse voltage is generated in the case of no 2DSL (**a**, **b**), non-canted (0°) 2DSL (**c**, **d**), or 30° canted 2DSL (**e**, **f**). Only in the trivial case of a 1D periodic modulation (**g**, **h**) a transverse voltage drop can be found.

Supplementary Figure 7

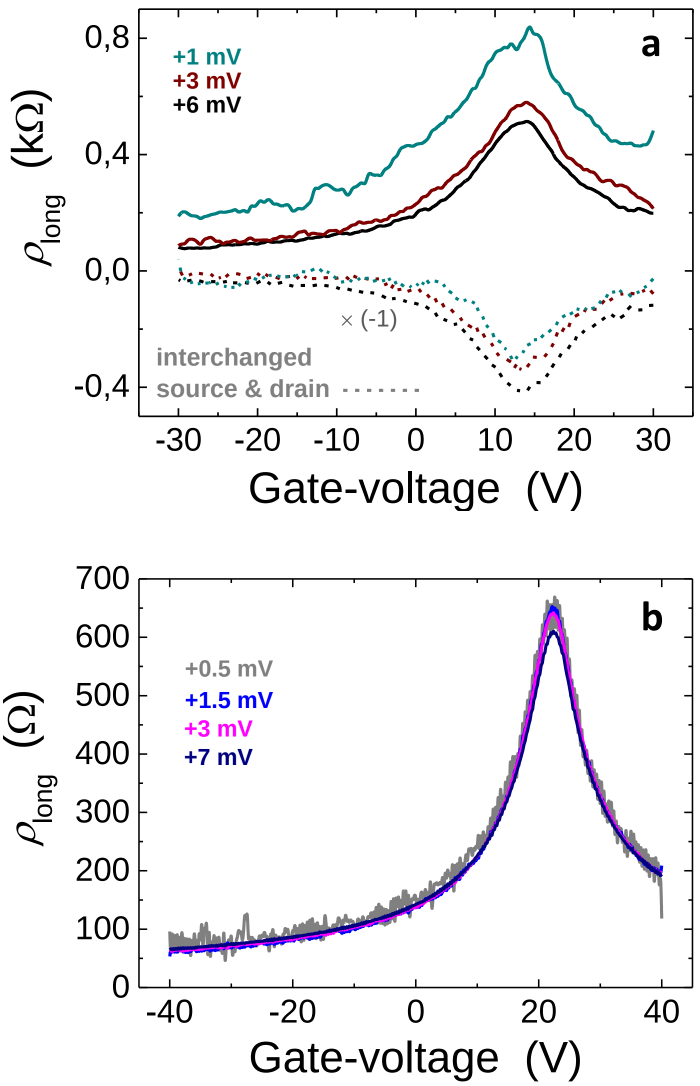

Supplementary Figure 7. Longitudinal resistivity ($\rho_{\rm long}$). (a) $\rho_{\rm long}$ of a $\alpha_{\rm 2DSL} = 30°$ Ti-defined and **(b)** $\rho_{\rm long}$ of a $\alpha_{\rm 2DSL} = 30°$ Pd-defined 2DSL sample. In both cases $\rho_{\rm long}$ some dependence with the source-drain voltage is present, however for the Pd-defined 2DSL this is much less pronounced.

Supplementary Figure 8

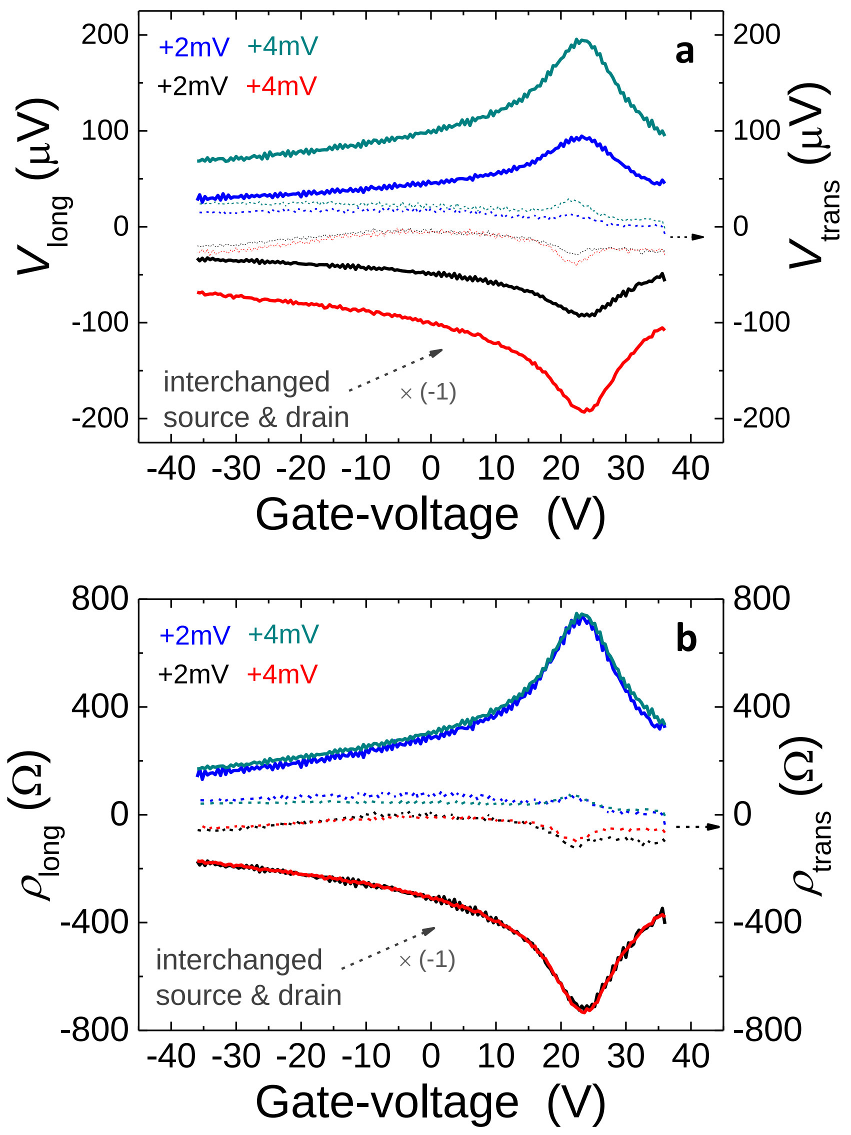

Supplementary Figure 8. Longitudinal and transverse voltages and resistivities for samples with non-canted dot array. (a) Longitudinal and transverse voltage (V_{long} and V_{trans}) of a sample with Ti-defined 2DSL (surface-hydrophobisised substrate) which is not canted relative to the overall current-direction for two source-drain-voltage magnitudes. The transverse component (dashed line) is more than an order of magnitude smaller than the longitudinal (full line) component. **(b)** Associated

longitudinal and transverse resistance (ρ_{long} and ρ_{trans}). The magnitude difference in the two components is even more prominent than for the corresponding voltages. In addition, both resistance components show no or hardly any dependence on the source-drain voltage which differs from all samples with canted 2DSL. Hence, the small residual transverse component in samples with non-canted 2DSLs is due to the technological lithographic imprecision in 2DSL alignment in the sample-production.

Supplementary Figure 9

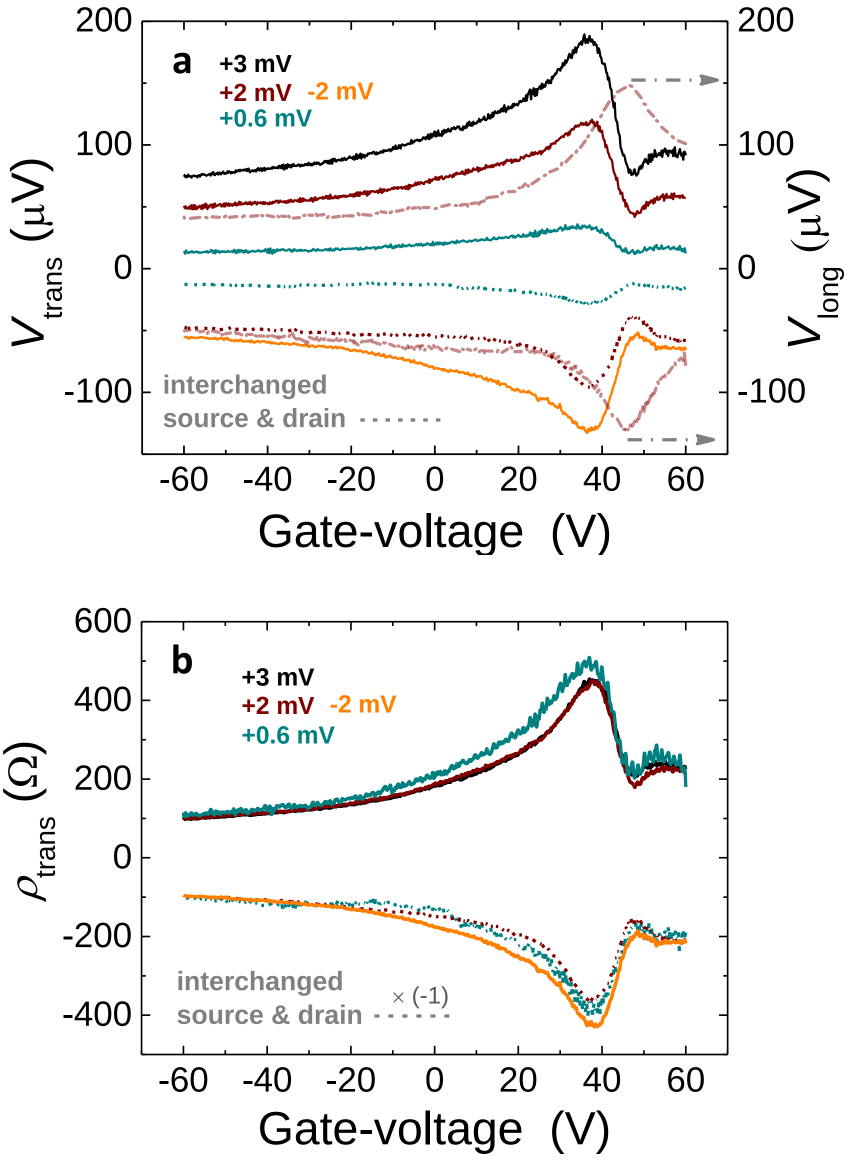

Supplementary Figure 9. Transverse voltages and resistivities for samples with canted dot array on a bare SiO_2 substrate. (a) V_{trans} of a sample with Ti-defined 2DSL (α_{2DSL} = 30°) but the substrate being bare SiO_2 for different DC source-drain-voltage magnitudes, directions and signs. The transverse-voltage effect is recovered proving that this effect is due to the presence of the canted 2DSL. V_{long} for +3 mV source-drain voltage is shown, too, (half-transparent lines), and is larger

than V_{trans}. This demonstrates a higher amount of randomly distributed localised charged scattering-centres induced by the bare SiO_2 substrate into the graphene. **(b)** The associated ρ_{trans} shows the same non-monotonous behaviour and variation with lower source-drain voltage as in all other samples.

Supplementary Figure 10

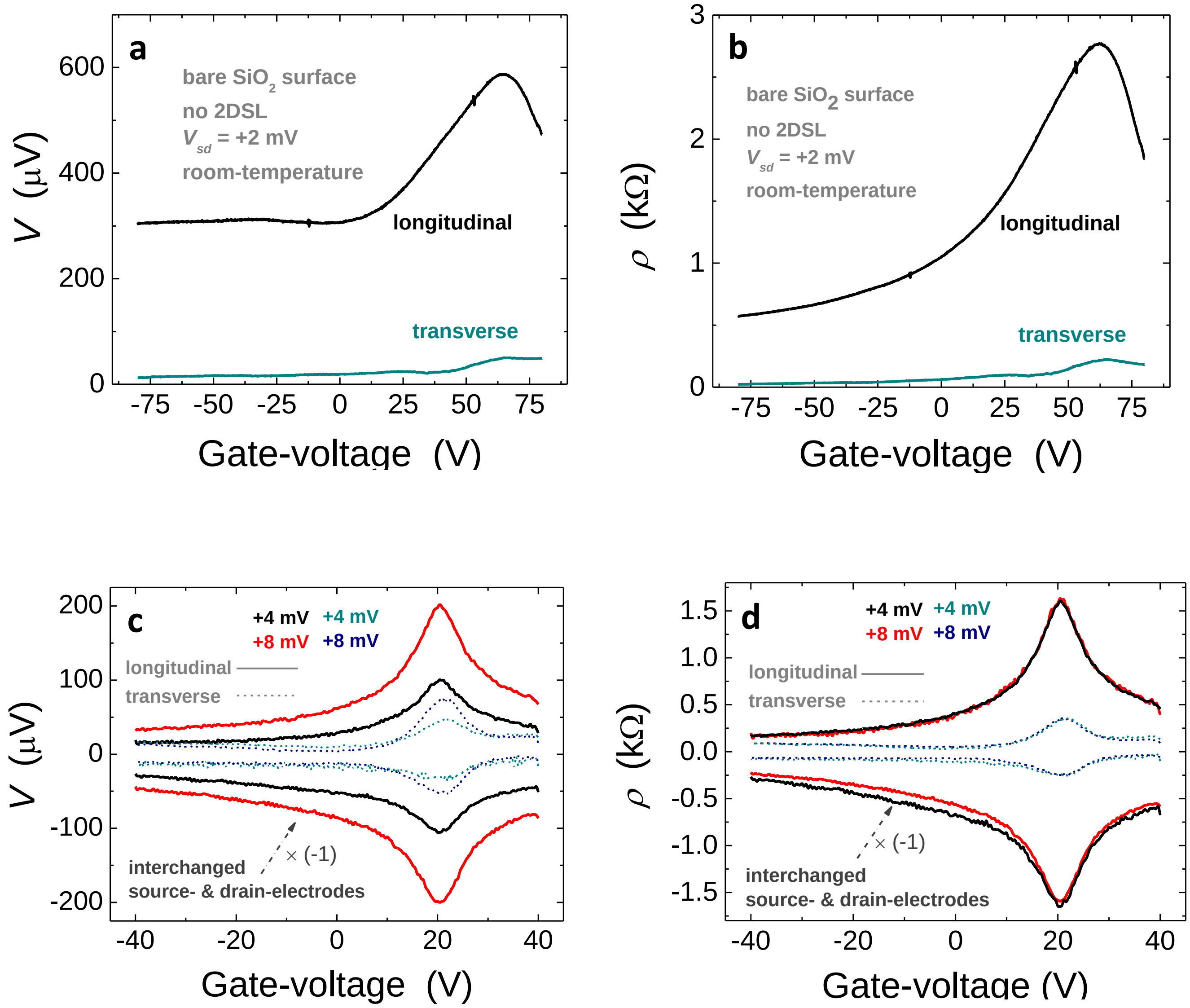

Supplementary Figure 10. Longitudinal and transverse voltages and resistivities for samples without dot array. (a) and **(b)** show the longitudinal and transverse voltage and resistance components for a sample without 2DSL and no hydrophobidised surface (bare SiO_2 surface). For both, voltage and resistance, the longitudinal components are more than one order of magnitude larger than the transverse ones. In addition, the transverse components show no non-monotonous

behaviour with well-developed and characteristic minimum and maximum as in all other samples with canted 2DSL. **(c)** and **(d)** show the longitudinal and transverse voltage and resistance components for a sample without 2DSL and hydrophobically rendered surface. The longitudinal components are again significantly larger than the transverse component as for the sample with bare SiO_2 surface in **a** and **b** and show no sign of any non-monotonous behaviour with a maximum and minimum. Most strikingly, neither the longitudinal nor the transverse resistance components have any dependence on the applied source-drain voltage. This is unambiguous proof that, as in **a** and **b**, all transverse components measured in samples without 2DSL are due to the technological limitations (resolution-limit) of the lithographic alignment of electrodes in the sample-production process.

Supplementary Figure 11

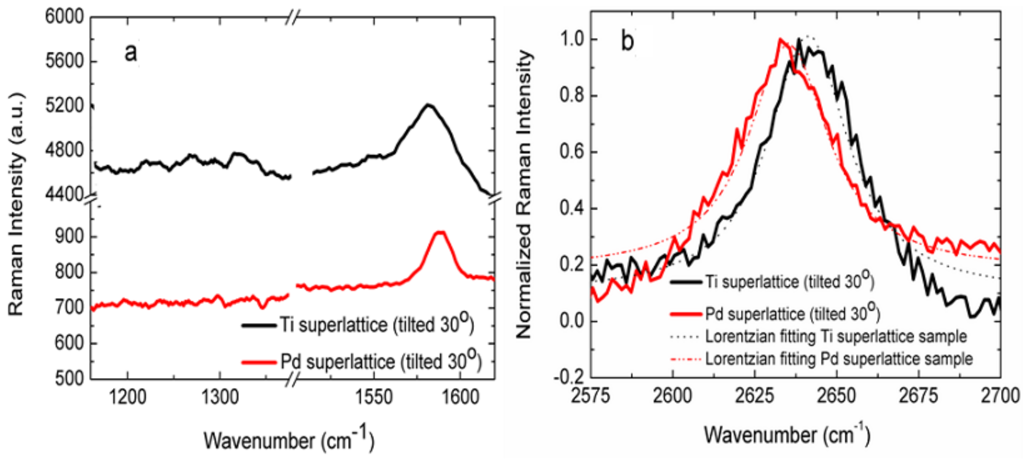

Supplementary Figure 11. Typical Raman spectra of the graphene samples with 30° canted Ti (black) and Pd (red) 2DSL (devices presented in Figures 2 and 3 main manuscript, respectively). Raman measurements are performed after patterning the 2DSL in order to evaluate the impact of the dots in the graphene (possible defects). **(a)** Spectra within the range [1100 cm^{-1} – 1650 cm^{-1}]. The D peak at ~1300 cm^{-1} is small compared to the G peak at ~1580 cm^{-1} for the Ti 2DSL and is absent in the Pd case. This demonstrates that the metallic 2DSL does not introduce a considerable amount of defects in graphene. **(b)** 2D peak at ~2650 cm^{-1} of both devices fitted with a single Lorentzian peak (dotted line) as a proof of the graphene monolayer [1].

Supplementary Tables

Supplementary Table 1

V_{CNP} (V)	Ti/Pd	n_{imp} (cm^{-2})	μ_{min} ($m^2V^{-1}s^{-1}$)	$\ell_{m,CNP}$ (nm)	$\ell_{m,10V}$ (nm)	T (K)	air/vacuum	FoM
14	Ti	1×10^{12}	1.2	140	180	293	vacuum	14.1
14	Ti	1×10^{12}	1.2	140	180	200	vacuum	16.3
21	Pd	1.5×10^{12}	0.8	115	140	293	vacuum	2.6
23	Ti	1.65×10^{12}	0.72	105	130	293	vacuum	2.1
25	Ti	1.8×10^{12}	0.66	100	120	293	air	2.2
28	Ti	2×10^{12}	0.6	93	115	293	vacuum	1.8
45	Ti	3.23×10^{12}	0.37	77	84	293	vacuum	1.2
45	Ti	3.23×10^{12}	0.37	77	84	200	vacuum	1.5

Supplementary Table 1. Summary of measurement and transport parameters. Charge neutrality point V_{CNP}, metal composing the dots, charge impurity density n_{imp}, mobility μ_{min}, Coulomb mean free path at the charge neutrality point (minimum mean free path) $\ell_{m,CNP}$, mean free path at 10 V away from the charge neutrality point $\ell_{m,10V}$, temperature, experimental environment and FoM of all the samples presented in Fig. 1f main text.

Supplementary Notes

Supplementary Note 1

As described in the experimental part of the main text, our system shows the following characteristics:

- Observation of a transverse voltage V_{trans} (ρ_{trans}) with a step-like feature for α_{2DSL} =30°.
- V_{trans} (ρ_{trans}) is present even under diffusive transport conditions.
- V_{trans} (ρ_{trans}) is not present for α_{2DSL} =0°.
- Step-like feature is inverted depending on the local shift in energy in graphene (*p*- or *n*-type).

The relativistic scattering formalism based on wavelike behaviour as demonstrated above is able to explain the transverse voltage generation and its characteristics. We show here that diffusive and non-relativistic numerical and analytical models cannot reproduce any of the observed experimental features.

First the diffusive and non-relativistic resistor network can be considered following a model proposed by Parish and Littlewood.[16] The model was originally created to investigate magneto-resistive effects in inhomogeneous conductors, here however only zero field calculations were carried out. In this model the conductor is represented by a network of four terminal resistors (discs). Two opposing sides, say left and right side, of the network are connected to a potential bank respectively while the contacts on the top and bottom are open. With this boundary conditions the potentials at the terminals of each resistor in the network is calculated using both Ohm's and Kirchhoff's current laws. Local scattering potentials (dopants) are

inserted as regions with different resistivity (black dots). For the transverse-voltage determination, the potential differences between opposing contacts on the top and bottom of the network are evaluated.

In a network consisting of 20 x 40 discs without an imposed superlattice of scattering-potentials (all resistors equal) the current flows directly from one potential bank to the other without the appearance of a transversal potential drop (see **Supplementary Figures 13a** and **b**).

The interesting case in our study is the imposed square 2D (scattering-)potential superlattice (2DSL) which is either oriented at 0° (see **Supplementary Figure 13c**) or at 30° (see **Supplementary Figure 13e**) relative to the current direction.

To keep the relative sizes similar as in the experiments in our 20 x 40 discs network, we **Supplementary Figures 11c** and **e**).

In the case of a non-canted (0°) 2DSL in **Supplementary Figure 11c** no transverse potential drop was found as shown in **Supplementary Figures 11d**. In fact, the potential-landscape is found to be identical as for the case of no 2DSL imposed (*cf*. **Supplementary Figures 11a** and **b**).

In our experiments we found for a 30° canted 2DSL a transverse voltage. Modelling this situation with the diffusive non-relativistic resistor network as shown in **Supplementary Figure 11e**, we find no trace of the appearance of a transverse voltage (see **Supplementary Figure 11f**). Again the corresponding potential-landscape is identical to the cases without 2DSL (**Supplementary Figures 11a** and **b**) and with non-canted (0°) 2DSL (**Supplementary Figures 11c** and **d**).

We emphasize that even for extreme ratios (up to four orders of magnitude) between the resistances of the black and white discs the calculation shows no transversal

potential differences. In addition, no transverse voltage difference is observe even in the case of using a larger network (50 x 100) or larger spacing between the local scattering potentials. Finally, we confirmed that moving from a squared canted 2DSL to the trivial case of a canted 1D periodic potential modulation we find a transverse voltage as was for the latter case previously demonstrated [17]. Our model is therefore fully consistent.

Another viewpoint is from a pure analytical perspective when a diffusive and non-relativistic resistivity tensor is considered. Within this framework, the need for the absence of V_{trans} in the previous resistor network model can be easily pinpointed. The most general form of the off-diagonal resistivity tensor in diffusive and periodically modulated non-relativistic systems including a rotation angle α_{2DSL} in the modulation potential is [17]

$$\rho_{\mathrm{trans}} = \rho_{\mathrm{H}} + \left(\rho_{\mathrm{par}} - \rho_{\mathrm{perp}}\right) \cdot \sin\alpha_{\mathrm{2DSL}} \cdot \cos\alpha_{\mathrm{2DSL}}$$

where the resistivities for the current flow parallel and perpendicular to the periodic potential are denoted by ρ_{par} and ρ_{perp}, respectively, and ρ_{H} is the Hall component which in our case is 0 as there is no magnetic field present. In our case of a square periodic potentials, however, $\rho_{\mathrm{par}} = \rho_{\mathrm{perp}}$, which leads to $\rho_{\mathrm{trans}} = 0$ regardless of the rotation angle. Therefore, no transverse signal can be present in such systems.

Supplementary Note 2

Longitudinal resistivity - The longitudinal resistivity was ρ_{long} was as usual determined by $\rho_{\text{long}} = \frac{V_{\text{long}}}{I_{\text{b}}} \cdot \frac{W}{L}$ for all types of samples where V_{long} is the (4-point longitudinal voltage) measured, I_{b} the simultaneously measured current traversing the sample, L the centre-to-centre separation of the voltage-probes and W the width of the sample.

For $\alpha_{\text{2DSL}} = 30°$ samples, the magnitude of ρ_{long} has some dependence on the applied source-drain voltage, however, does not show any shift in gate-voltage with source-drain-voltage. In contrast, for $\alpha_{\text{2DSL}} = 0°$ samples (see **Supplementary Note 2**) basically neither a dependence of the magnitude of ρ_{long} nor any shift of the curve on the applied bias voltage is found.

Indeed, for $\alpha_{\text{2DSL}} = 30°$ some ρ_{long} magnitude dependence on the applied source-drain voltage is even expected as cascaded Mie scattering supports ballistic remainders even under otherwise diffusive conditions (main text). The magnitude of variation of ρ_{long} in $\alpha_{\text{2DSL}} = 30°$ samples is therefore linked to the charge-carriers mean free path. The Ti sample in **Supplementary Figure 2a** shows a more pronounced magnitude variation on source-drain voltage than the Pd sample in **Supplementary Figure 2b** because it has a larger mean-free-path [18,19] (*cf*. **Supplementary Table 1**).

Supplementary Note 3

To compare quantitatively devices with canted periodic potentials (2DSLs) on different substrates and measured under different conditions (Fig. 1f main text), we calculate the mobility of the samples in order to estimate their mean free path. Our entire devices are always diffusive at the measured temperatures. Within a first approximation, the overall mobility of any of our devices would be composed of three contributions: the intrinsic contribution due to phonons, contribution due to charged impurities and contribution due to the imposed periodic potentials (circular dots). We note that we are mainly interested in the charged impurity contribution to the mobility since for a constant temperature the other two factors are equal for the devices we want to compare in Fig. 1f main text (*i.e.* devices with periodic potentials and α_{2DSL} = 30°). Furthermore, the phonon contribution to mobility even at room temperature is estimated to give a mobility value of ~20 m^2 V^{-1} s^{-1} [9], implying that this factor is not the main contributor in our case (we have a much lower overall mobility). Regarding the circular metallic dots, it is predicted [3] and experimentally proved [10] that the scattering cross section of a single dot with size $k_{\mathrm{F}}D$ >1 (as in our samples) is comparable to that of a single impurity (k_{F} is the Fermi wavevector). These studies imply that circular dots are not the main mobility contributor in our case either since our dot density n_{dot}~1 x 10^{-9} cm^{-2}, which is a much smaller value than the impurity concentration n_{imp}, calculated from the V_{CNP} [10]. When the Fermi energy $E_{\mathrm{F}} = V_{\mathrm{CNP}}$, the mobility value due to charge impurities is the minimum for the entire gate-voltage sweep [10,12-14] and it can be estimated following the equation $\mu_{\mathrm{min}} = \frac{K}{n_{\mathrm{imp}}}$ [10-14].

The constant K has been measured to be between 5 x 10^{15} V^{-1} s^{-1} [12] and 1.2 x 10^{16}

V^{-1} s^{-1} [15]. For our estimations, we take 1.2 x 10^{16} V^{-1} s^{-1} which is the more conservative value to estimate the mobility in order to show that our devices work within the diffusive regime. In **Supplementary Table 1** the charge neutrality point, metal composing the dots, charge impurity density, mobilities, Coulomb mean free path at the charge neutrality point (minimum mean free path), mean free path at 10 V away from the charge neutrality point, temperature, measuring environment and FoM of all the samples presented in Fig. 1f main text are shown.

Supplementary Note 4

The persistence of the non-monotonic transverse voltage also in the case of canted Ti-2DSL samples without surface hydrophobisation is illustrated in **Supplementary Figures 4a** and **4b** where V_{trans} and ρ_{trans}, respectively, are shown for several source-drain-voltage magnitudes, signs and directions for a graphene sample on bare SiO_2. The associated V_{long} (half-transparent) for +3 mV source-drain voltage is given, too. Clearly, V_{trans} exhibits qualitatively the identical non-monotonous behaviour as samples with surface-hydrophobisation. This is further proof that the transverse-voltage is due to the existence of a canted 2DSL imposed on the graphene, that is, not a substrate effect. The dependence of V_{trans} with source-drain-voltage magnitude is less pronounced in comparison with samples with surface-hydrophobisation (FoM in these devices is lower, see Fig. 1f main text). This indicates the presence of more randomly distributed localised charged scatterers induced by the bare SiO_2 than in the samples with surface-hydrophobisation.

Supplementary Note 5

The voltages and resistances for samples without 2DSL show no non-monotonous transverse voltage with a step-like feature in it. This is demonstrated in **Supplementary Figures 5a** and **5b** (sample with bare SiO_2 surface), and **5c** and **5d** (sample with surface hydrophobidisation). For samples with a bare SiO_2 surface both the longitudinal voltage- and resistance-component are more than an order of magnitude higher than the transverse one. In addition the transverse components show no sign of a well-developed and characteristic maximum and minimum behaviour like observed for samples with canted 2DSL (*cf*. Fig. 2 and 3b main text; Fig. 4). Instead the residual transverse components follow the longitudinal ones as expected if this component is solely due to the limit in the lithographical alignment precision in the production of the samples.

The same qualitative behaviour is found for the sample with hydrophobically-rendered surface (**Supplementary Figures 5c and d**). Strikingly, *longitudinal and transverse resistances do not depend on the applied source-drain voltages*. This is in contrast to all samples with canted 2DSL. This proves again that the transverse components measured in samples without 2DSL are due to the limit in the lithographical alignment precision in the sample-production process.

Supplementary Note 6

The impact of surface hydrophobisation on non-canted Ti-2DSL samples is illustrated in **Supplementary Figure 3a** where V_{trans} (dotted line) and V_{long} (solid line) for two source-drain voltages and directions are shown. The magnitude of V_{trans} is one to two orders lower than V_{long} and their FoM < 0.1, in clear contrast to samples with canted lattice. This difference is even more prominent when transforming the data into ρ_{trans} and ρ_{long} (**Supplementary Figure 3b**). In particular, there is basically no dependence of ρ_{long} and ρ_{trans} on the source-drain voltage in contrast to all samples with canted 2DSL. Hence, in samples with no canted 2DSL, the V_{trans} (ρ_{trans}) signal is merely a residual of the limit in the lithographical alignment precision of the 2DSL in the production of the samples.

Supplementary Methods

Supplementary Method 1

Computational approach to model the transverse voltage generation and the step-feature in the transverse voltage - We first give detailed physical arguments for the transversal voltage $V_{\rm trans}$ generation in canted relativistic 2D potential superlattices. Then, the gate dependence of $V_{\rm trans}$ is modelled, showing an excellent qualitative agreement with the experiments.

Any transversal voltage generation $V_{\rm trans}$ is ultimately caused by a spatially imbalanced charge-carrier density n across the graphene device. To prove this imbalance in n in our canted superlattice devices, there is a need to consider both the scattering through a single local circular potential and the periodic spatial arrangement of a multiple of these potentials.

Regarding the scattering by a single circular potential, it has to be considered that the presence of metal atoms on top of graphene introduces a local shift in the charge-neutrality point $V_{\rm CNP}$ in this material by a quantity $V_{\rm pot}$ [2,3]. If the device is appropriately gated, metallic impurities therefore create local *p-n* junctions (PNJs) within the monolayer [2,3]. Theoretically, the elastic scattering of relativistic carriers in graphene due to an individual circular PNJ has been recently addressed in Refs. 5-8. The low-energy charge-carrier dynamics is calculated through the Dirac Hamiltonian

$$H = -i\nabla\sigma + V_{\rm pot}\Theta(R-r) \quad \text{(Eq.1)}$$

under the assumptions of a sharp potential on the scale of the Fermi wavelength ($\lambda_{\rm F} >> d_{\rm pot}$) and no intervalley scattering ($d_{\rm pot} >> a$). $\lambda_{\rm F}$ is the Fermi wavelength

outside the junction, d_{pot} is the characteristic length-scale in which the scattering potential varies, a is the graphene lattice-constant, $\sigma = (\sigma_{x,}\sigma_y)$ are the Pauli matrices, R is the radius of the circle, and Θ is the Heaviside function. The scattering problem is solved by expanding the incident plane wave in eigenfunctions in polar coordinates $\{r,\phi\}$ and matching the reflected and transmitted waves at $r = R$ to satisfy the continuity of the wavefunction [3-6].

This formalism is analogous to the description of Mie scattering in optics [6]. Known phenomena from optics appear in graphene in new guise to satisfy the absence of backscattering dictated by Klein tunnelling [3-6].

We are interested in the charge-carrier density n outside the metallic dot and the scattered current-density j, which are given by $n = \psi^*\psi$, and $j = \psi^*\sigma\psi$, respectively, where $\psi = \psi^{(\text{in})} + \psi^{(\text{ref})}$. $\psi^{(\text{in})}$ and $\psi^{(\text{ref})}$ are the incoming and reflected wave functions [4]:

$$\psi^{(\text{in})} = \frac{1}{\sqrt{2}}\begin{pmatrix} e^{ikx} \\ \alpha e^{ikx} \end{pmatrix} = \frac{1}{\sqrt{2}}\sum_{m=-\infty}^{m=\infty} i^{m+1}\begin{pmatrix} -iJ_m(k_F r)e^{im\phi} \\ \alpha J_{m+1}(k_F r)e^{i(m+1)\phi} \end{pmatrix} \quad \text{(Eq.2)}$$

$$\psi^{(\text{ref})} = \frac{1}{\sqrt{2}}\sum_{m=-\infty}^{m=\infty} i^{m+1} a_m^{(r)}\begin{pmatrix} -iH_m^{(1)}(k_F r)e^{im\phi} \\ \alpha H_{m+1}^{(1)}(k_F r)e^{i(m+1)\phi} \end{pmatrix} \quad \text{(Eq.3)}$$

Here, k_{F} is the wave vector outside the circular potential, J_m and $H_m^{(1)}$ are the Bessel's and Hankel's function of the first kind, respectively. $\alpha = \text{sgn}(E_{\text{F}})$ and $\alpha' = \text{sgn}(E_{\text{F}} - V_{\text{pot}})$ are band indices outside and inside the metallic dotted region and account for the four possible junction types: *nn'n*, *npn*, *pnp*, and *pp'p*. The reflection scattering coefficient is given in terms of the size parameter $\rho = k_{\text{F}}R$ as:

$$a_m^{(r)} = -\frac{J_{m+1}(N\rho)J_m(\rho) - \alpha\alpha' J_m(N\rho)J_{m+1}(\rho)}{J_{m+1}(N\rho)H_m^{(1)}(\rho) - \alpha\alpha' J_m(N\rho)H_{m+1}^{(1)}(\rho)} \quad \text{(Eq.4)}$$

In this equation, N (equivalent to the modulus of the refractive index) is given by $N = \left|V_{\mathrm{pot}} - E_{\mathrm{F}}\right| / \left|E_{\mathrm{F}}\right|$.

For a large diameter dot compared to the Fermi-wavelength of the Dirac-fermion, $\lambda_{\mathrm{F}} >> 2R$, as it is in our case for $\left|V_{\mathrm{gate}} - V_{\mathrm{D}}\right|$> 1 V, the scattering shows features known from geometrical optics. The boundary of the gated region acts as a lens focusing the charge-carrier beam generating two caustics which coalesce in a cusp inside the PNJ [4, 5, 7]. It can be observed (Fig. 6) that for any incoming plane wave in the x direction, n is symmetric in the transverse (y) direction, that is, with respect to the x axis going through the centre of the dot. Therefore, the carrier density along the transverse direction is spatially balanced in the case of the scattering by a single circular metallic dot. This spatially balanced charge-carrier density after scattering on one single dot therefore cannot account for producing a V_{trans} across the device.

In addition, when $\lambda_{\mathrm{F}} < 2R$, (as in our experiments for $\left|V_{\mathrm{gate}} - V_{\mathrm{D}}\right|$> 1 V), the scattered current $j = (j_x, j_y)$ in the far-field shows a peaked forward scattering [5,6]. The far-field condition can be therefore considered as being already well-fulfilled at distances > $2R$ away from the circular potential centre, that is, j has a dominating j_x component there [4].

As shown by the foregoing calculations, the scattering by a single circular potential can be analytically determined by the generalization of the partial wave method [3-6] similar to Mie scattering [4]. In the 2DSL case, the scattering problem gets more

complex depending on the additional parameter of the superstructure: the 2DSL tilting angle α_{2DSL} measured w.r.t. the overall current direction (*i.e.* propagation direction of incoming Dirac-fermion wavefunction). To extend the solution for the scattering by a single circular potential (dot) to the square 2DSL of circular potentials, we applied the transfer matrix method (TMM). These types of methods are extensively used for solving scattering problems in periodic (identical) potentials [8]. Within this formalism, we can connect the simple single dot scatterers as building block to generate square 2DSLs (Fig. 7) and determine the total transfer matrix by simple multiplication.

As discussed beforehand, within the far-field regime (*i.e.* distances $> 2R$ away from the circular potential centre; see also Ref. 4), a single circular potential acts on an incoming plane wave in the x direction in such a way that it generates a symmetric redistribution of the (charge-carrier) probability density in the transverse (y) direction. In addition, in terms of overall current, in the far-field limit only the current component in positive x direction is non-vanishing. These conditions can be expressed as follows:

$$\psi_L^{(+)} = \psi^{(\mathrm{in})} = \frac{1}{\sqrt{2}}\begin{pmatrix} e^{ik_F x} \\ \alpha e^{ik_F x} \end{pmatrix} \quad \text{(Eq.5)}$$

$$\psi_R^{(+)} = \psi^{(\mathrm{out})} = F(y)\psi^{(\mathrm{in})} = \frac{F(y)}{\sqrt{2}}\begin{pmatrix} e^{ik_F x} \\ \alpha e^{ik_F x} \end{pmatrix} \quad \text{(Eq.6)}$$

$$\psi_R^{(-)} = \psi_L^{(-)} = 0 \qquad \text{(Eq. 7)}$$

where the function $F(y)$ is in general complex valued and normalised. We will see later that it represents the same redistribution as demonstrated in section (i) of the charge-carrier density $n^{(\mathrm{out})}$ along the transverse direction (y axis) with respect to

the incoming density $n^{(\mathrm{in})}$ as already visualised in Fig. S8. The conditions of Eqs. 5-7 are fulfilled by considering the following transfer matrix M :

$$M = \begin{pmatrix} F(y) & 0 \\ 0 & 0 \end{pmatrix} \text{ (Eq. 8)}$$

Consequently, $n^{(\mathrm{out})}$ is given by

$$n^{(\mathrm{out})}(y) = \psi^{(\mathrm{out})^*}\psi^{(\mathrm{out})} = F^* F = \left|F^2\right| \qquad \text{(Eq. 9)}$$

A further condition on $F(y)$ is imposed by the fact that the transversal component of the scattered current, j_y, has to vanish in the far-field [4] as was already found in section (i). In the case of two dot-scattering, values of $F(y)$ are taken numerically after the scattering of each dot at a fixed far-field position (x = $4R$ in the case of Figs. 1c and d, main text).

The $|F^2|$ values plotted along the y-axis at a constant position $x > 2R$ are effectively the far-field scattering intensity which we use for further evaluation as follows.

We calculate the electronic (that is, basically the charge-carrier) density redistribution due to the scattering on two consecutive potentials within the TMM framework. The only required condition is that the 2DSL dot-to-dot distance $d_{\mathrm{dot-dot}}$ lies within the far-field (*i.e.* $d_{\mathrm{dot-dot}} > 2R$ as expressed before). We emphasise that our experimental conditions of the investigated devices are within the far-field limit since they have $d_{\mathrm{dot-dot}} = 7R$.

The existence of a transverse voltage can already be shown for the most simple case of only two cascaded circular potentials separated by the distance $d_{\mathrm{dot-dot}}$ under the angle α_{2DSL}. We found that $F(y)$ and in particular $\left|F^2\right|$ (*i.e.* the redistributed

electronic density) in the far-field limit can be well approximated by the analytical 5th-order Bessel-function

$$J_5(k_\mathrm{F}(y-y_0)). \qquad \text{(Eq. 10)}$$

For the two device geometries (dots aligned and canted with respect to the current direction), we find, using $\alpha_\mathrm{2DSL}=30°$ and $d_\mathrm{dot-dot}=350$ nm which are the parameters in our samples which we investigated experimentally:

- **<u>Non-cascaded case</u>.** If the centres of the two circular potentials are aligned with respect to the incoming current, that is, α_2DSL= 0, and therefore $y_0=d_\mathrm{dot-dot}\sin(\alpha_\mathrm{2DSL})=0$, then the electronic density $n^{(out)}\propto J_5(k_\mathrm{F}y)^4$, that is, the far-field scattering intensity is symmetric with respect to the transverse (y) direction (see Fig. 9, black curve, and Fig. 3a in main text, blue curve).
- **<u>Cascaded case</u>.** If the centres of the two circular potentials are not aligned respect the incoming current, that is, $\alpha_\mathrm{2DSL}\neq 0$, and therefore $y_0=d_\mathrm{dot-dot}\sin(\alpha_\mathrm{2DSL})\neq 0$, then the electronic density is found to be $n^{(out)}\propto J_5(k_\mathrm{F}y)^2 J_5(k_\mathrm{F}(y-d_\mathrm{dot-dot}\sin(\alpha_\mathrm{2DSL})))^2$, that is, the far-field scattering intensity is not spatially symmetric with respect to the transverse (y) direction. That is, an intensity imbalance establishes comparing the positive and the negative y -axis (see Fig. 9 and Fig 3a in main text red curves).

Therefore, our results clearly demonstrate that V_trans is effectively generated by the imbalanced electronic density-redistribution introduced by cascading circular scattering potentials as we realised them in our experiments.

We emphasise, that for a given current direction the two potentials (dots) case can be seen as new unit cell from which a larger 2DSL with arbitrary many dots can be

built. If these two-potential unit cells have canted potentials and are arranged into a periodic square lattice, as in our devices, a transverse voltage V_{trans} will be generated.

Regarding the step-like feature in the transverse voltage, that is, the qualitative dependence of V_{trans} on the Fermi energy (gate-voltage V_{gate}) of the massless Dirac-particles in the vicinity of the charge-neutrality point, V_{CNP}, the same formalism as described in the previous paragraphs can be used. In here, we focus not only on the existence of an electronic density imbalance, but also its variation with V_{gate}. For an evaluation, it is sufficient to consider the far-field scattering intensity (*cf*. Fig. 9) along the y-axis of the (cascaded) two-dot unit cell. Integrating the intensity independently for positive and negative y and taking the difference of these values provides an intensity imbalance and is a direct measure for the deviation of the incident electronic wave due to the canted geometry. Fig. 3a in the main text shows clearly that the intensity imbalance is maximal at a Fermi energy close to V_{pot} (Fig. 3a main text top); minimal at a Fermi energy close to the V_{CNP} (Fig. 3a main text middle) and takes an inbetween value away from these two points (Fig 3a main text bottom). Therefore, already the model for two dots considered in previous paragraphs is able to reproduce the characteristic step-like feature present in the experiments.

More precisely, the step-like feature can be traced back to Mie scattering occurring in single dots (circular potentials). To show that the non-monotonous step-like dependence of V_{trans} on the electronic energy (V_{gate}) is genuine to Mie scattering, we evaluate the gate dependence of the scattering of a Dirac-fermion wave at a single dot in the far-field ($r > 2R$) using Eqs. 1-4 following the coordinate system depicted

in Fig. 10 and assuming an incoming electronic plane wave travelling in the x direction. As aforementioned, these systems are characterized by the absence of back-scattering and a peaked forward scattering [5-8], thus, we consider only transmitted and scattered probabilities in the far-field limit. We emphasise that this calculation is similar to the one leading to Fig. 9, able to produce the far-field scattering intensity along the y-axis for the two-dot case. Furthermore, we note that this approach implicitly implies the multiple Klein-phenomenon occurring at the circular potential [5-8]. For a given V_{gate} we consider as the 'transmitted probability', T, the probability for a Dirac-fermion to be within $y \in [-R, R]$ in the far-field (*i.e.* at any $x > 2R$). Meanwhile, the 'scattered probability' S is the probability for a Dirac-fermion to be within $|y| > R$ in the far-field (*i.e*. at any $x > 2R$). This is illustrated for Dirac electrons in Fig. 10. These calculations in the far-field limit can be done through the following numerical integrations:

$$T(V_{\mathrm{gate}}) = \int_{y=-R}^{y=R} n(V_{\mathrm{gate}}, x = C)dy \quad \text{(Eq. 11)}$$

$$S(V_{\mathrm{gate}}) = \int_{y=-C}^{y=-R} n(V_{\mathrm{gate}}, x = C)dy + \int_{y=R}^{y=C} n(V_{\mathrm{gate}}, x = C)dy \quad \text{(Eq.12)}$$

where the $C \geq 2R$ was chosen to ensure the far-field condition (in our case we chose $C = 2R$ and $C = 3R$ obtaining similar results).

The dark-blue curve in the top panel of Fig. 3b (main text) shows the calculated gate dependence ($V_{\mathrm{gate}} - V_{\mathrm{CNP}} \in$ [-40 V, +40 V]) of the total scattered far-field intensity (S-regions in Fig. 10, representing basically the differential cross section) of a single circular dot with potential $V_{\mathrm{pot}} = -0.12$ eV (Ti-like dot).

When increasing the gate voltage, it shows first a maximum around -10 V, a sudden decrease from -10 V until 7 V and a constant dependency afterwards. In contrast, the dark-blue curve in the bottom panel of Fig. 3b (main text) shows the result for a single dot with potential $V_{\text{pot}} = +0.06$ eV (Pd-like dot) which is qualitatively inverted relative to the gate-voltage (energy) compared to the top-panel. However, in both cases a minimum is observed a the charge neutrality point $(\Delta V_{\text{gate}} = 0)$. Our calculations reproduce very well the step-like feature found in both types of our samples. That is, the maximum in V_{trans} corresponds to the maximum in the scattering probability which is achieved when $E_{\text{F}} = V_{\text{pot}}$. Then, V_{trans} decreases until $E_{\text{F}} = V_{\text{CNP}}$, which corresponds to a decrease in scattering probability. Therefore, V_{pot} is directly linked to the appearance of the relative maxima and the minima of V_{trans} and to the multiple Klein tunnelling phenomena occurring in circular potentials, which is implicitly considered in the here applied model [3,4].

The experimental transverse voltages in Fig. 3b main text were taken at 0.5 (black), 1 (red), 1.5 (blue), and 5 mV (dark yellow) for the Pd-2DSL sample, and for the Ti-2DSL sample at 1 (black), 3 (wine), and 6 mV (green).

Supplementary References

1. Ferrari, A. C., Meyer, J. C., Scardaci, V., Casiraghi, C., Lazzeri, M., Mauri, F., Pisanec, S., Jiang, D., Novoselov, K. S., Roth, S. & Geim, A. K. Raman spectrum of graphene and graphene layers. *Phys. Rev. Lett*. **97,** 187401 (2006).

2. McCreary, K. M., Pi, K., & Kawakamib, R. K. Metallic and insulating adsorbates on graphene. *Appl. Phys. Lett.* ***98***, 192101 (2011).

3. Katsnelson, M. I., Guinea, F. & Geim, A. K. Scattering of electrons in graphene by clusters of impurities. *Phys. Rev. B* **79**, 195426 (2009).

4. Heinisch, R. L., Bronold, F. X. & Fehske, H. Mie scattering analog in graphene: Lensing, particle confinement, and depletion of Klein tunneling. *Phys. Rev. B* **87,** 155409 (2013).

5. Cserti, J., Pályi, A. & Péterfalvi, C. Caustics due to a negative Refractive Index in Circular Graphene p-n junctions. *Phys. Rev. Lett.* **99**, 246801 (2007).

6. Guinea, F. Models of Electron Transport in Single Layer Graphene. *J. Low. Temp. Phys.* **153**, 359-373 (2008).

7. Cheianov, V. V., Fal’ko, V., Altshuler, B. L. The focusing of electron flow and Veselago lens in graphene p-n Junctions. *Science* **315**, 1252-1255 (2007).

8. Monsoriu, J. A., Villatoro, F. R., Marín, M. M., Pérez, J. & Monreal, L. Quantum fractal superlattices. *Am. J. Phys*. **74**, 831-836 (2006).

9. Wang, L. *et al*. One-Dimensional Electrical Contact to a Two-Dimensional Material. *Science* **342**, 614-617 (2013).

10. McCreary, K. M. *et al*. Effect of cluster formation on graphene mobility. *Phys. Rev. B* **81**, 115453 (2010).

11. Wu, J. S., & Fogler, M. M., Scattering of two-dimensional massless Dirac electrons by a circular potential barrier. *Phys. Rev. B* **90**, 235402 (2014).

12. Chen, J. H. *et al*. Charged-impurity scattering in graphene. *Nature Phys*. **4**, 377-381 (2008).

13. Shishir, R. S. & Ferry, D. K. Intrinsic mobility in graphene. *J. Phys. Cond. Mat.* **21**, 232204 (2009).

14. Hwang, E. H., Adam, S. & Das Sarma, S. Carrier transport in two-dimensional graphene layers. *Phys. Rev. Lett.* **98**, 186806 (2007).

15. Chen, B. *et al*. How good can CVD-grown monolayer graphene be? *Nanoscale* **6**, 15255-15261 (2014).

16. Parish, M. M. & Littlewood, P. B. Non-saturating magnetorresistance in heavily disordered semiconductors. *Nature* **426**, 162-165 (2003).

17. Schlösser, T. *et al*. Corrugation-induced transverse voltage in a lateral superlattice. *Phys. Rev. B* **51**, 10737-10742 (1995).

18 Sivan, U., Heiblum, M., Umbach, C.P. & Strickman, H. Electrostatic electron lens in the ballistic regime. *Phys. Rev. B* **41**, 7937-7940 (1990).

19 Spector, J., Stormer, H. L., Baldwin, K. W., Pfeiffer, L. N. & West, K. W. Electron focusing in two-dimensional systems by means of an electrostatic lens. *Appl. Phys. Lett*. **56**, 1290-1292 (1990).
